# Short term efficacy of nebulized beclomethasone in mild-to-moderate wheezing episodes in pre-school children

**DOI:** 10.1186/1824-7288-37-39

**Published:** 2011-08-22

**Authors:** Alberto Papi, Gabriele Nicolini, Attilio L Boner, Eugenio Baraldi, Renato Cutrera, Leonardo M Fabbri, Giovanni A Rossi

**Affiliations:** 1 Department of Respiratory Diseases, Research Centre on Asthma and COPD, University of Ferrara, Ferrara, Italy; 2 Medical Department, Chiesi Farmaceutici, Parma, Italy; 3 Department of Pediatrics, University of Verona, Verona, Italy; 4 Department of Pediatrics, Unit of Allergy and Respiratory Medicine, University of Padova, Padova, Italy; 5 Department of Pediatrics, Ospedale Pediatrico Bambino Gesù, Roma, Italy; 6 Department of Respiratory Diseases, University of Modena, Modena, Italy; 7 Department of Pediatrics, Ospedale Gaslini, Genova, Italy

## Abstract

**Background:**

Few data are available on the usefulness of short term treatment with low-medium dose of inhaled corticosteroids (ICS) in pre-school children with wheezing exacerbations.

**Methods:**

To compare the efficacy of one week treatment with 400 μg b.i.d. nebulized beclomethasone dipropionate (BDP), plus nebulized 2500 μg prn salbutamol (BDP group), versus nebulized b.i.d. placebo, plus nebulized prn 2500 μg salbutamol (placebo group), a post-hoc analysis was performed on data obtained in 166 pre-school children with multiple-trigger wheezing, recruited during an acute wheezing episode.

**Results:**

The percentage of symptom-free days (SFDs) was significantly higher in the BDP group (54.7%) than in the placebo group (40.5%; p = 0.012), with a 35% relative difference. Day-by-day analysis showed that the percentage of SFDs was already higher in the BDP group after 2 days (7.4%), the difference reaching statistical significance at day 6 (12.3%; p = 0.035). Cough score was also reduced in the BDP group (0.11) as compared with the placebo group (0.39; p = 0.048), the difference reaching statistical significance after 5 days of treatment (0.18 and 0.47 respectively; p = 0.047). The mean number of nebulizations per day of prn salbutamol was lower in the BDP group as compared to the placebo group (0.26 and 0.34, respectively), but the difference was not significant (p = 0.366). There were no differences in positive effects of BDP treatment between children with and without risk factors for asthma.

**Conclusions:**

A 1-week treatment with nebulized BDP and prn salbutamol is effective in increasing SFDs and improving cough in children with wheezing, providing a clinical rationale for the short term use of ICS in episodic wheeze exacerbations in pre-school children.

**Trial Registration:**

ClinicalTrials.gov (NCT00497523)

## Background

Wheezing is a common symptom in pre-school aged children. Epidemiological studies have demonstrated that approximately 30% of children have at least 1 episode of wheezing before the age of 3, with a cumulative prevalence of wheezing of 50% at the age of 6 [1,2]. Most of these wheezing episodes are induced by viral upper respiratory tract infections (RTI), which recur frequently in this age group, and the great majority of young children will have only transient symptoms with no subsequent increased risk of asthma or allergy later in life [1-3].

Recurrent pre-school wheezing is a very common clinical problem, with important implications since it may be associated in developed countries with a remarkable impact on the healthcare budget [4]. Rates for wheezing-related emergency room visits and hospitalizations are highest among pre-school children, reflecting not only the specific morbidity in this population, but also the lack of conclusive evidence on the efficacy of available treatments/strategies for these acute events [4-6]. To date, the best treatment for children with intermittent wheezing remains controversial.

Recently, a variety of evidence-based international guidelines on the management of wheezing disorders in pre-school children have been published [3,6-10]. For persistent wheezing in children under the age of 5, low dose inhaled corticosteroids (ICS) is recommended as the preferred controller therapy, with leukotriene modifiers as an alternative. Systematic reviews of controlled trials evaluating the efficacy of ICS in infants/pre-schoolers with wheeze or asthma showed that, in those with frequent symptoms and/or with risk factors for asthma, ICS therapy improves a variety of relevant outcomes, including lung function and symptom-free days (SFDs), wheeze/asthma exacerbation and need for systemic glucocorticosteroids. In these studies, the beneficial effect was independent of age, atopic condition, type of ICS, mode of delivery and treatment duration [3,11,12]. This is in agreement with the results of a randomized controlled trial we recently published, in which 276 pre-school children (1-4 years of age) with a history of recurrent wheezing, recruited during an acute wheezing episode not requiring hospitalization or systemic ICS, were randomly assigned to 3-month nebulized treatments with: a) beclomethasone dipropionate (BDP) b.i.d, plus prn (pro re nata) salbutamol; b) BDP/salbutamol combination prn or c) prn salbutamol [13]. This trial showed a higher percentage of SFDs in the subgroup on regular nebulized BDP, as compared with prn salbutamol. However there were no differences between regular BDP and the prn combination subgroups on the primary outcome measure (percentage of SFDs) as well as on several secondary outcomes, such as symptom scores, use of relief medication and exacerbation frequency [13].

Systematic reviews and two recent reports have concluded that episodic high-dose inhaled glucocorticosteroids may provide some benefit in episodic (viral) wheeze [11,14-16] but, because of possible side effects (reduced height) [16], the use of high-dose intermittent steroids in this age group requires careful consideration [3,16].

Surprisingly, in contrast with the efficacy shown in medium- long-term treatments, the efficacy of short-term treatment with low-medium dose ICS is poorly documented in pre-school children with wheezing symptoms.

Here we report the results of a post-hoc analysis, performed in our aforementioned study [13], to evaluate whether low dose nebulized BDP (400 μg b.i.d), plus prn salbutamol, was superior to nebulized prn salbutamol in improving symptoms in the first week of treatment in pre-school children with a history of recurrent wheezing, recruited during an acute wheezing episode.

## Methods

A detailed description of the study design, including screening and recruitment procedures and statistical analysis, has been reported in detail elsewhere [13] and will only be briefly summarized.

Pre-school children (ages 1-4), with a documented history of at least 3 episodes of wheeze or asthma in the 6 months preceding the visit, evaluated as outpatients for exacerbation of respiratory symptoms were screened and characterized [13]. In addition to the three episodes in the previous 6 months, the included children had a history of frequent wheeze, induced by other triggers than viral respiratory tract infection [3]. Nineteen pediatric specialist care units were involved in this randomized, multicenter, double blind, parallel-group, placebo-controlled trial. Exclusion criteria were: a) history of severe exacerbations requiring systemic glucocorticoids; b) chest infection or hospitalization due to asthma in the previous 4 weeks; c) treatment with inhaled glucocorticoids or methyl-xanthine during the previous 4 weeks; d) treatment with oral glucocorticoids in the previous 8 weeks. Eligible children entered a 2-week prospective open run-in period during which they received only 2500 μg prn nebulized salbutamol for symptom relief. Patients entered the double-blind treatment phase if they had wheeze and/or cough, and/or shortness of breath, and/or required relief medication on at least 7 days of the 2-week run-in period. The study was performed in accordance with the Declaration of Helsinki and Good Clinical Practice guidelines. Independent ethics committees approved the study protocol, patient information sheet, and consent forms. A parent or guardian of each patient provided written informed consent. Parents/guardians were trained: a) to correctly use the nebulizer; b) to keep daily records of their child's symptoms, recording scores for wheeze, cough, and shortness of breath on diary cards, using a four-point rating scale ranging from 0 (no symptoms) to 3 (disabling symptom) [17]; c) to record the number of occasions during the night they were woken up because of the child's asthma symptoms which required rescue medication for symptom relief.

Children were stratified in two groups on the basis of the presence or absence of risk factors for developing persistent asthma [18]: (1) atopic dermatitis or eczema; (2) asthma in a first-degree relative; (3) blood eosinophilia > 4% demonstrated in the previous 6 months. A 2:1 treatment-to-placebo randomization ratio was used to expose fewer patients to placebo.

In the original study [13], 3 treatments were tested in this population over 12 weeks:

a) BDP (Clenil per Aerosol, Chiesi Farmaceutici, Parma, Italy) 400 μg/vial, one vial b.i.d., plus salbutamol (Ventmax, Chiesi Farmaceutici, Parma, Italy) 2500 μg/vial, one vial prn, or b) placebo one vial b.i.d., plus fixed combination of BDP and salbutamol (Clenil Compositum, Chiesi Farmaceutici, Parma, Italy), 800 μg BDP + 1600 μg salbutamol/vial, one vial prn, or c) placebo one vial b.i.d. plus salbutamol 2500 μg/vial, one vial prn. All drugs were delivered with the same nebulizer (Clenny aerosol; Medel, Parma, Italy) using a tight-fitting face mask.

In the current post-hoc analysis we compare the efficacy of regular BDP plus prn salbutamol (BDP group) vs. regular placebo plus prn salbutamol (placebo group) on clinical outcomes captured in patients' diaries during the first week of treatment.

Groups were compared in terms of percentage of SFDs at the end of 1-week treatment using an ANCOVA model with percentage of SFDs as dependent variable and treatment, country and subgroup as fixed effects and baseline (run-in) as covariate. A day-by-day analysis of the percentage of SFDs was also performed using the same ANCOVA model.

Moreover, a comparison between groups within each subgroup of the percentage of SFDs at the end of 1-week treatment was performed using the same ANCOVA model with also the subgroup*treatment interaction as fixed effect.

The proportion of patients without asthma symptoms during the first week of treatment was compared between groups by means of a logistic regression model with presence of symptoms during the first week as dependent variable, treatment, country, subgroup and percentage of SFDs during run-in as independent variables.

Each of the three symptom scores was also analyzed by means of a mixed model for repeated measures with score at each day as dependent variable, treatment, country, subgroup, day, treatment*day interaction and mean value of symptoms during run-in as independent variables. All tests were considered as explorative.

## Results

One hundred sixty-six pre-school children, 98 males and 68 females, (between ages of 1-4) were included in the post-hoc analysis: 110 were (64 (58.2%) males) in the BDP group and 56 (34 (60.7%) males) in the placebo group. The BDP group and the placebo group had similar baseline characteristics (table 1).

**Table 1:** Baseline characteristics

	Beclomethasone (BDP group) (N = 110)	Salbutamol (Placebo group) (N = 56)
Male, n (%)	64 (58.2)	34 (60.7)
Risk factors for asthma, n (%)	57 (51.8)	30 (53.6)
Age, yr	2.35 ± 0.81	2.29 ± 0.78
Weight, kg	15.4 ± 2.82	14.9 ± 2.74
Height, cm	97.5 ± 9.19	96.0 ± 9.61
Duration of wheezing, yr	1.59 ± 0.87	1.43 ± 0.82
Symptom-free days, %	21.4 ± 18.3	25.9 ± 21.0
Daily coughing score	1.43 ± 0.71	1.35 ± 0.80
Daily Salbutamol use, n^a^	0.49 ± 0.48	0.42 ± 0.35

Values of symptom-free days, daily coughing score and daily salbutamol use are mean of the 2-week run-in period ± SD. Beclomethasone = beclomethasone dipropionate; ^a ^number of nebulizations per day.

**Figure 1:**
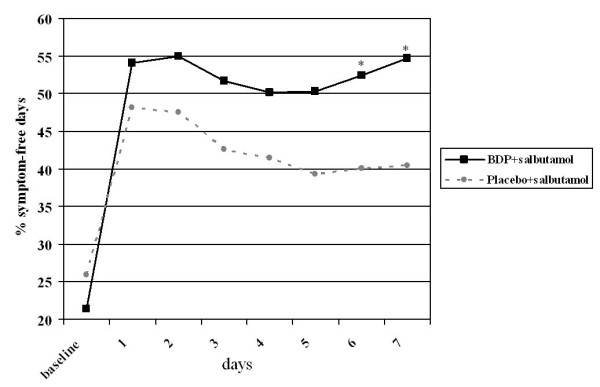
Percentage of symptom-free days in the first week of treatment; each day represents the cumulative percentage of symptom-free days in the preceding period; on day 1 the data is relative to the % of symptom-free patients; * p < 0.05 between groups.

**Figure 2:**
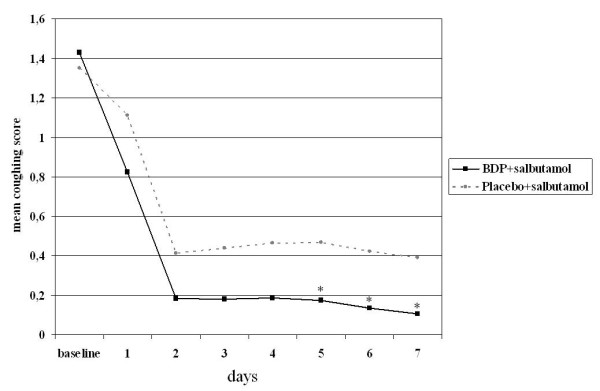
Mean coughing score in the first week of treatment; each day represents the cumulative mean coughing score; * p < 0,05 between groups.

The adjusted mean percentage of SFDs (the study primary outcome [13]) at the end of the 7-day-treatment was significantly higher in the BDP group (54.7%) than in the placebo group (40.5%) (p = 0.012). The proportion of patients without asthma symptoms during the first week of treatment was significantly higher in the BDP group than in the placebo group (odds ratio 2.65; 95% CI: 1.08, 6.51, p = 0.033). Day-by-day analysis showed that the adjusted mean percentage of SFDs was already higher after 2 days of treatment in the BDP group (55.0%) as compared with the placebo group (47.5%) with a 7.4% difference, the difference between groups reaching statistical significance after 6 days of treatment (52.4% in BDP group, 40.1% in placebo group with a 12.3% difference, 95% CI of difference: 0.88, 23.8; p = 0.035; Figure 1). At the end of the 7-day-treatment, the adjusted mean coughing score was also lower in the BDP group (0.11) compared to the placebo group (0.39) with a difference of -0.29 (95% CI of difference: -0.568, -0.002; p = 0.048); the between-group difference reached statistical significance as early as day 5 of treatment (difference between BDP and placebo group is -0.29; p = 0.047; Figure 2). No significant differences were found between the two groups with respect to other secondary outcomes such as nocturnal awakenings, wheezing score, shortness of breath score and symptom score (p > 0.05, all comparisons, data not shown) although all were numerically in favour of BDP. The positive effects of BDP treatment on percentage of SFDs were similar in children with and without risk factors for asthma, the difference in adjusted mean percentage of SFDs in the first week of treatment being 2.06% (p = 0.75). Notably, the difference between BDP and placebo was significant in the subgroup of children with risk factors for asthma, with 19.06% more SFDs as compared to placebo (p = 0.014) but it was not significant in the subgroup without risk factors (p = 0.257) despite 9.24% more SFDs.

The adjusted mean number of nebulisations per day of prn salbutamol was lower in the BDP group as compared to the placebo group (0.26 and 0.34, respectively) although the difference was not significant (p = 0.366).

## Discussion

The results of this post-hoc analysis show the efficacy of a short treatment with nebulized BDP in pre-school children with a mild-to-moderate wheezing episode not requiring hospitalization or systemic corticosteroids.

Previous studies on intermittent treatment with inhaled glucocorticoids in pre-school wheeze exacerbations led to conflicting results [3,11,12]. Three studies suggested that initiating ICS therapy at the early signs of episodic wheezing/asthma exacerbation in pre-school children does not result in reduction in oral corticosteroid use [19-21]. These studies, however, were small and used different time points for intervening with medication, thus limiting interpretation of results. Also a study performed in 411 infants, enrolled at one month of age to evaluate the effects of ICS initiated after a three-day episode of wheezing, did not demonstrate any short-term benefit during the acute manifestation or on the progression from episodic to persistent wheezing [22]. Lack of effect might be related to the specific pathophysiology of wheezing episodes present in young children.

More recently, Bacharier and co-workers, in a randomized, double-blind, placebo-controlled trial compared the efficacy of 7 day treatment with either budesonide inhalation suspension (1 mg twice daily), montelukast (4 mg daily), or placebo in addition to salbutamol at the early signs of RTI in pre-school children with moderate-to severe intermittent wheezing [15]. No differences in the proportion of episode-free days, oral corticosteroid and health care use, quality of life, or linear growth was observed in the three treatment groups during the 12-month trial. However, during RTI, administration of budesonide and of montelukast led to significant reduction of episode severity as compared with conventional therapy with reductions in trouble breathing and interference with activity scores. The efficacy was most evident during the peak symptom period of the first 48 hours of illness and in children with positive asthma predictive indices [15,18].

Our results extend these observations showing the efficacy of low dose nebulized BDP, detectable a few days after the beginning of treatment. In contrast with Bacharier's study [15], we did not see any significant difference in the response to treatment between children with positive or negative asthma predictive indices either in the original 12-week trial or in this 1-week analysis. This is in agreement with previous reports where no differences in response to ICS were reported by pre-school children with and without risk factors for asthma, including atopic condition, sensitization to inhaled allergens, and eczema [12,23,24]. Differences in the study design and in the patient population may explain the discrepancy between Bacharier's study [15] and ours. Nevertheless, we found that the increase in the percentage of SFDs after 1 week of BDP treatment, as compared to placebo, was significant only for the subgroup of children with risk factors.

Finally, an interesting hypothesis is that the episodic use of an ICS (e.g. BDP) during acute RTI could decrease an important source of future respiratory morbidity, in these children, i.e. airway inflammation and remodeling, leading to a medium-long-term down regulation of the severity of the subsequent exacerbations [13].

The percentage of SFDs is a frequently used measure for asthma control and reflects the multiple components of asthma disease burden. Although often informative in comparing the effects of long-term controller medications for asthma in patients with chronic symptoms, it has been shown to be also sufficiently sensitive to detect treatment effects among children with an episodic disorder, such as severe intermittent asthma [15]. The demonstration of the efficacy of a short treatment with BDP in the present trial is of interest even from a safety point of view, considering that we detected no systemic effects of the 12 weeks treatment in the original trial by measuring cortisol at the beginning and end of treatment [13]. The systemic side effects of corticosteroids are considered negligible in short term therapies but this is in contrast with recent findings. A study evaluating the immune response of children aged three to 17, arriving at the emergency room with an asthma attack showed an immune suppression in children receiving oral corticosteroids [25]. In light of this new evidence, the use of nebulized rather than oral corticosteroids may be preferable even for short term treatments.

The limit of the present study is that the post-hoc analyses were based only on the data of the first week of a 3-month clinical trial and P-values of these analyses were unadjusted for multiplicity.

## Conclusions

In conclusion, this study shows the short term efficacy of a one week nebulized BDP plus salbutamol as needed treatment on SFDs and coughing in young children with wheezing. Our findings, along with recent pharmacological reports showing the rapid onset of action of ICS [26,27] could provide a clinical rationale for the short term use of ICS in episodic wheeze, one of the most frequent recurrent respiratory disorders in pre-school children.

## Competing interests

AP, ALB, EB, RC, LMF and GAR declare no conflict of interest. GN is a Chiesi Farmaceutici employee.

## Authors' contributions

AP, GN and GAR contributed to designing the study, data analysis and writing the manuscript; ALB, EB, RC and LMF contributed to data analysis and interpretation and writing the manuscript. All authors read and approved the final manuscript.
